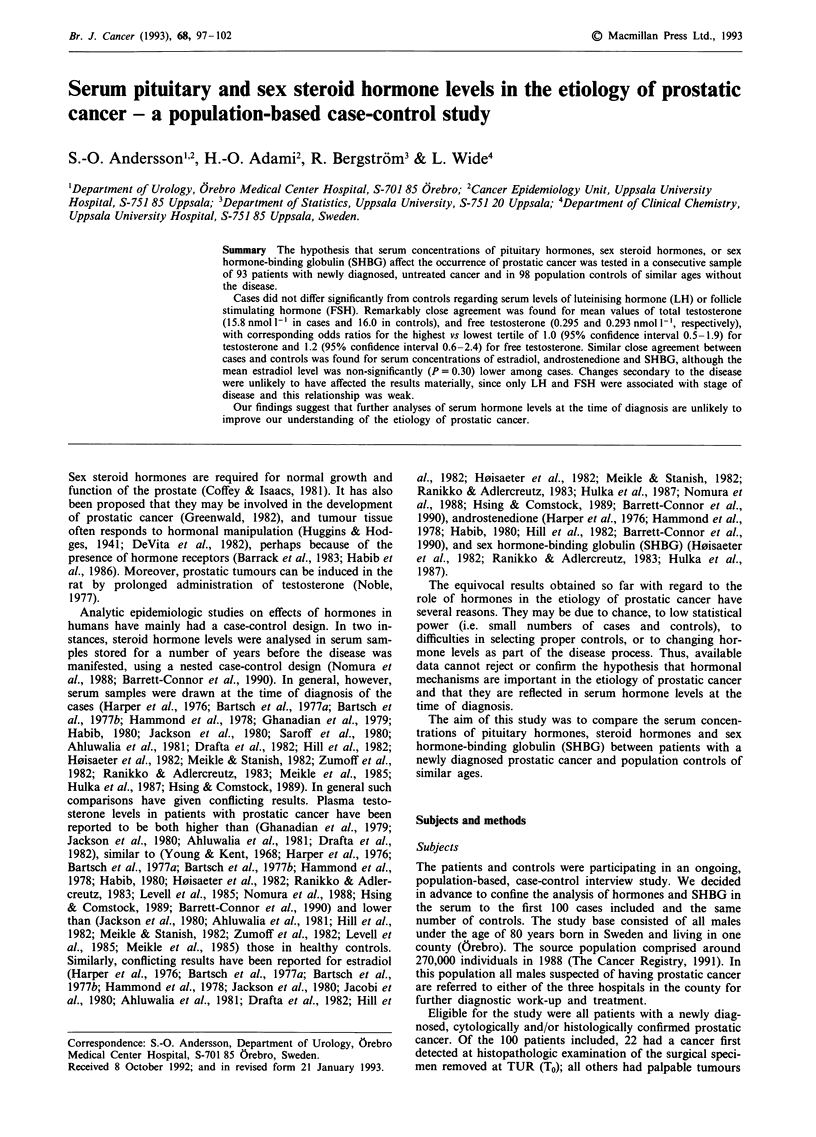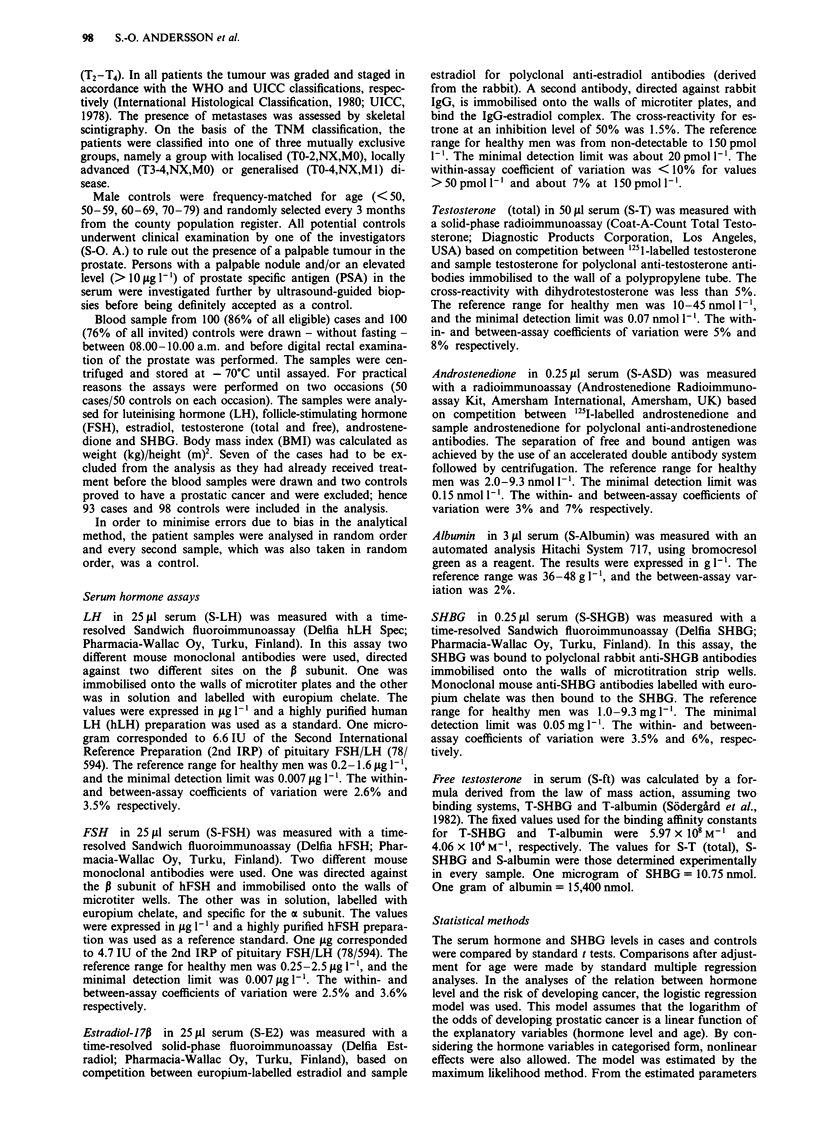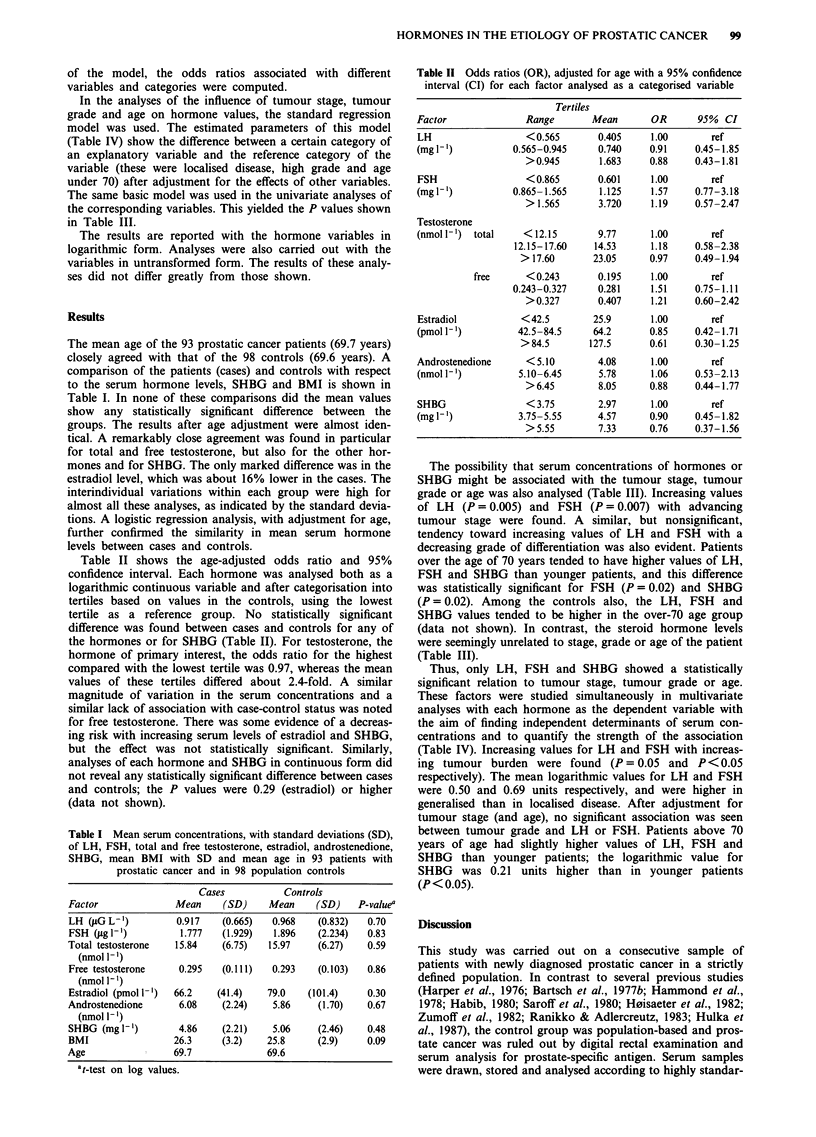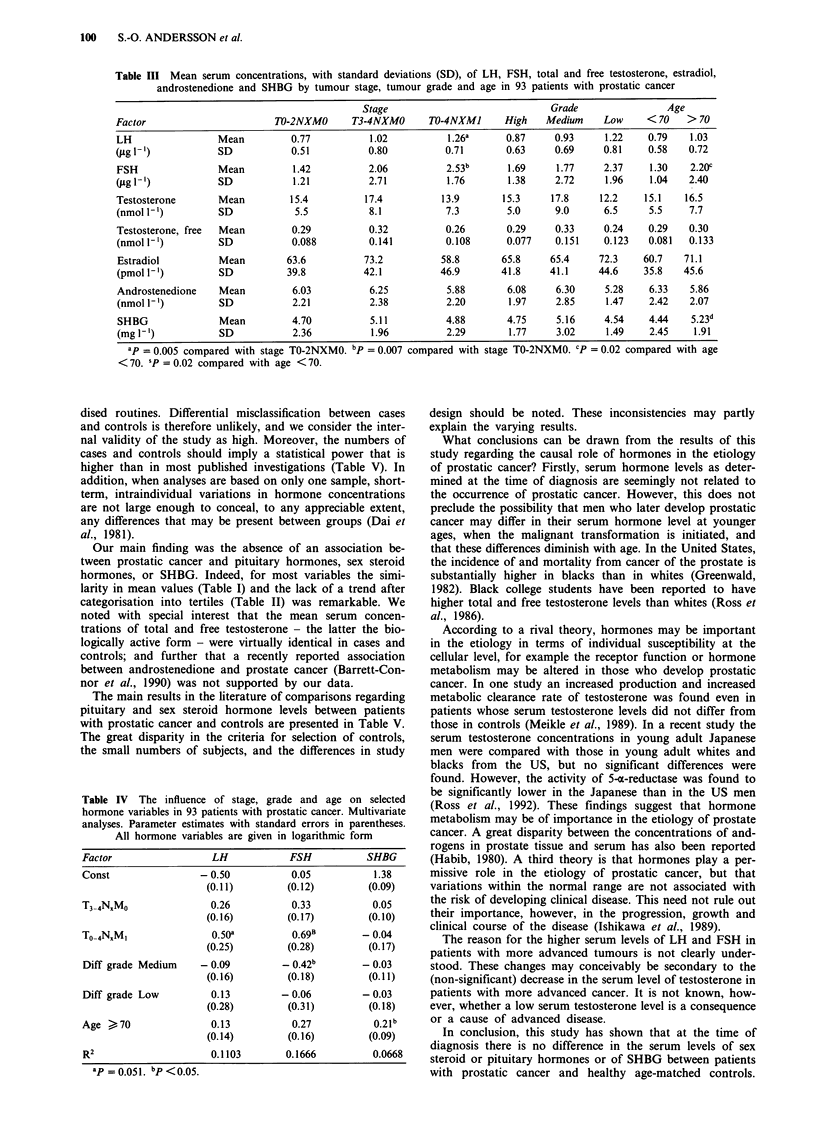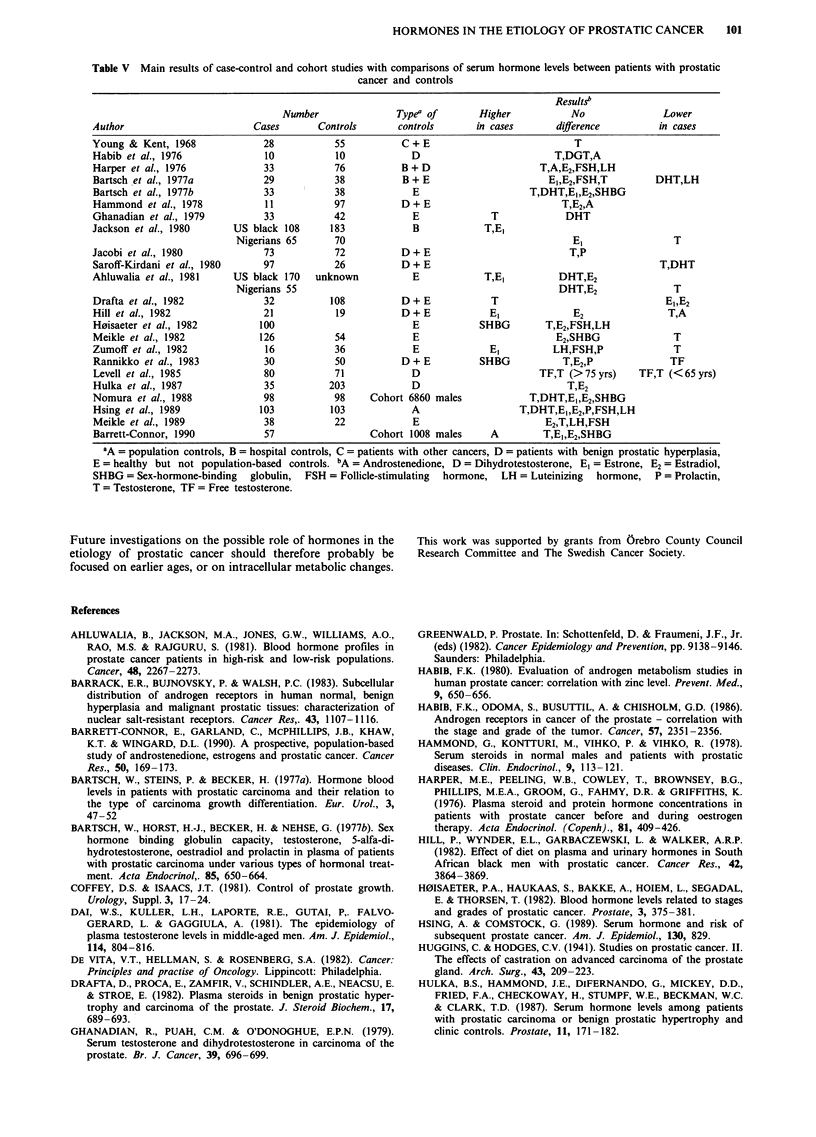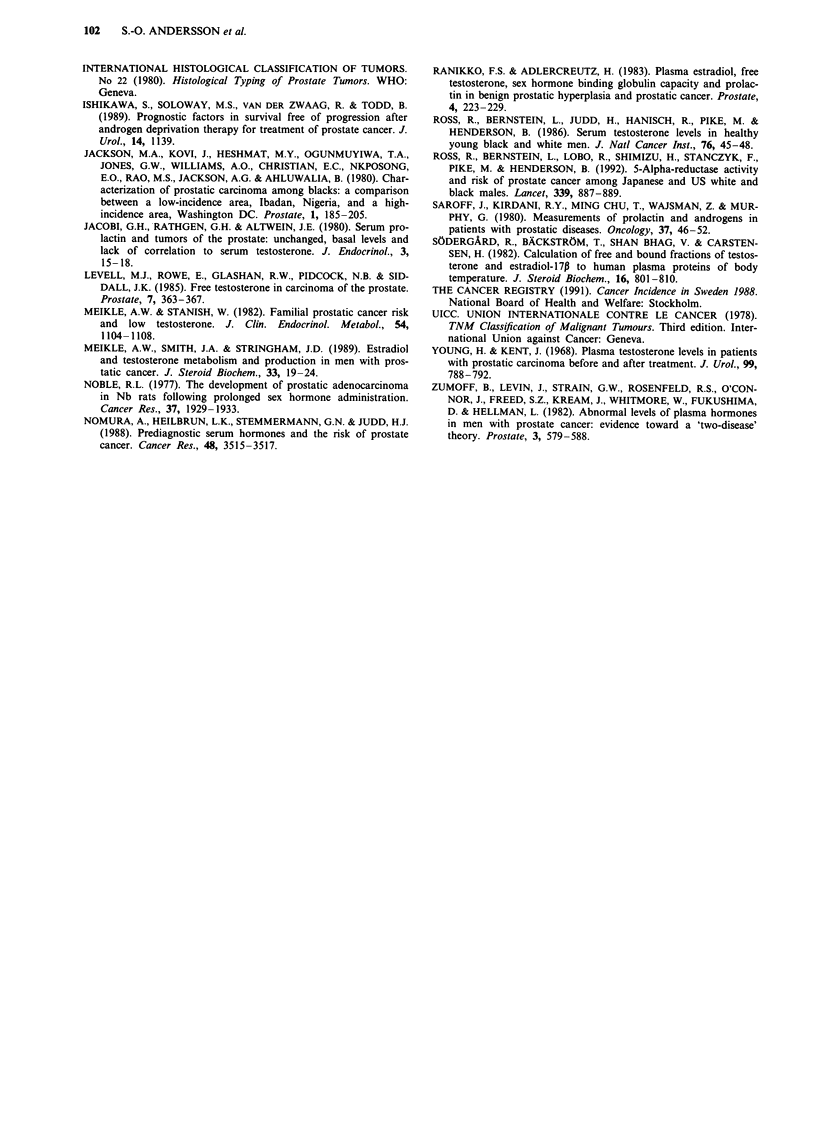# Serum pituitary and sex steroid hormone levels in the etiology of prostatic cancer--a population-based case-control study.

**DOI:** 10.1038/bjc.1993.293

**Published:** 1993-07

**Authors:** S. O. Andersson, H. O. Adami, R. Bergström, L. Wide

**Affiliations:** Department of Urology, Orebro Medical Center Hospital, Sweden.

## Abstract

The hypothesis that serum concentrations of pituitary hormones, sex steroid hormones, or sex hormone-binding globulin (SHBG) affect the occurrence of prostatic cancer was tested in a consecutive sample of 93 patients with newly diagnosed, untreated cancer and in 98 population controls of similar ages without the disease. Cases did not differ significantly from controls regarding serum levels of luteinising hormone (LH) or follicle stimulating hormone (FSH). Remarkably close agreement was found for mean values of total testosterone (15.8 nmol l-1 in cases and 16.0 in controls), and free testosterone (0.295 and 0.293 nmol l-1, respectively), with corresponding odds ratios for the highest vs lowest tertile of 1.0 (95% confidence interval 0.5-1.9) for testosterone and 1.2 (95% confidence interval 0.6-2.4) for free testosterone. Similar close agreement between cases and controls was found for serum concentrations of estradiol, androstenedione and SHBG, although the mean estradiol level was non-significantly (P = 0.30) lower among cases. Changes secondary to the disease were unlikely to have affected the results materially, since only LH and FSH were associated with stage of disease and this relationship was weak. Our findings suggest that further analyses of serum hormone levels at the time of diagnosis are unlikely to improve our understanding of the etiology of prostatic cancer.


Br. J. Cancer (1993), 68, 97–102 © Macmillan Press Ltd., 1993

### Serum pituitary and sex steroid hormone levels in the etiology of prostatic cancer – a population-based case-control study

S.-O. Andersson[1,2], H.-O. Adami[2], R. Bergström[3] & L. Wide[4]

[1]*Department of Urology, Örebro Medical Center Hospital, S-701 85 Örebro;* [2]*Cancer Epidemiology Unit, Uppsala University Hospital, S-751 85 Uppsala;* [3]*Department of Statistics, Uppsala University, S-751 20 Uppsala;* [4]*Department of Clinical Chemistry, Uppsala University Hospital, S-751 85 Uppsala, Sweden.*

**Summary** The hypothesis that serum concentrations of pituitary hormones, sex steroid hormones, or sex hormone-binding globulin (SHBG) affect the occurrence of prostatic cancer was tested in a consecutive sample of 93 patients with newly diagnosed, untreated cancer and in 98 population controls of similar ages without the disease.

Cases did not differ significantly from controls regarding serum levels of luteinising hormone (LH) or follicle stimulating hormone (FSH). Remarkably close agreement was found for mean values of total testosterone (15.8 nmol $l^{-1}$ in cases and 16.0 in controls), and free testosterone (0.295 and 0.293 nmol $l^{-1}$, respectively), with corresponding odds ratios for the highest *vs* lowest tertile of 1.0 (95% confidence interval 0.5–1.9) for testosterone and 1.2 (95% confidence interval 0.6–2.4) for free testosterone. Similar close agreement between cases and controls was found for serum concentrations of estradiol, androstenedione and SHBG, although the mean estradiol level was non-significantly ($P = 0.30$) lower among cases. Changes secondary to the disease were unlikely to have affected the results materially, since only LH and FSH were associated with stage of disease and this relationship was weak.

Our findings suggest that further analyses of serum hormone levels at the time of diagnosis are unlikely to improve our understanding of the etiology of prostatic cancer.

Sex steroid hormones are required for normal growth and function of the prostate (Coffey & Isaacs, 1981). It has also been proposed that they may be involved in the development of prostatic cancer (Greenwald, 1982), and tumour tissue often responds to hormonal manipulation (Huggins & Hodges, 1941; DeVita *et al.*, 1982), perhaps because of the presence of hormone receptors (Barrack *et al.*, 1983; Habib *et al.*, 1986). Moreover, prostatic tumours can be induced in the rat by prolonged administration of testosterone (Noble, 1977).

Analytic epidemiologic studies on effects of hormones in humans have mainly had a case-control design. In two instances, steroid hormone levels were analysed in serum samples stored for a number of years before the disease was manifested, using a nested case-control design (Nomura *et al.*, 1988; Barrett-Connor *et al.*, 1990). In general, however, serum samples were drawn at the time of diagnosis of the cases (Harper *et al.*, 1976; Bartsch *et al.*, 1977*a*; Bartsch *et al.*, 1977*b*; Hammond *et al.*, 1978; Ghanadian *et al.*, 1979; Habib, 1980; Jackson *et al.*, 1980; Saroff *et al.*, 1980; Ahluwalia *et al.*, 1981; Drafta *et al.*, 1982; Hill *et al.*, 1982; Høisaeter *et al.*, 1982; Meikle & Stanish, 1982; Zumoff *et al.*, 1982; Ranikko & Adlercreutz, 1983; Meikle *et al.*, 1985; Hulka *et al.*, 1987; Hsing & Comstock, 1989). In general such comparisons have given conflicting results. Plasma testosterone levels in patients with prostatic cancer have been reported to be both higher than (Ghanadian *et al.*, 1979; Jackson *et al.*, 1980; Ahluwalia *et al.*, 1981; Drafta *et al.*, 1982), similar to (Young & Kent, 1968; Harper *et al.*, 1976; Bartsch *et al.*, 1977*a*; Bartsch *et al.*, 1977*b*; Hammond *et al.*, 1978; Habib, 1980; Høisaeter *et al.*, 1982; Ranikko & Adlercreutz, 1983; Levell *et al.*, 1985; Nomura *et al.*, 1988; Hsing & Comstock, 1989; Barrett-Connor *et al.*, 1990) and lower than (Jackson *et al.*, 1980; Ahluwalia *et al.*, 1981; Hill *et al.*, 1982; Meikle & Stanish, 1982; Zumoff *et al.*, 1982; Levell *et al.*, 1985; Meikle *et al.*, 1985) those in healthy controls. Similarly, conflicting results have been reported for estradiol (Harper *et al.*, 1976; Bartsch *et al.*, 1977*a*; Bartsch *et al.*, 1977*b*; Hammond *et al.*, 1978; Jackson *et al.*, 1980; Jacobi *et al.*, 1980; Ahluwalia *et al.*, 1981; Drafta *et al.*, 1982; Hill *et al.*, 1982; Høisaeter *et al.*, 1982; Meikle & Stanish, 1982; Ranikko & Adlercreutz, 1983; Hulka *et al.*, 1987; Nomura *et al.*, 1988; Hsing & Comstock, 1989; Barrett-Connor *et al.*, 1990), androstenedione (Harper *et al.*, 1976; Hammond *et al.*, 1978; Habib, 1980; Hill *et al.*, 1982; Barrett-Connor *et al.*, 1990), and sex hormone-binding globulin (SHBG) (Høisaeter *et al.*, 1982; Ranikko & Adlercreutz, 1983; Hulka *et al.*, 1987).

The equivocal results obtained so far with regard to the role of hormones in the etiology of prostatic cancer have several reasons. They may be due to chance, to low statistical power (i.e. small numbers of cases and controls), to difficulties in selecting proper controls, or to changing hormone levels as part of the disease process. Thus, available data cannot reject or confirm the hypothesis that hormonal mechanisms are important in the etiology of prostatic cancer and that they are reflected in serum hormone levels at the time of diagnosis.

The aim of this study was to compare the serum concentrations of pituitary hormones, steroid hormones and sex hormone-binding globulin (SHBG) between patients with a newly diagnosed prostatic cancer and population controls of similar ages.

### Subjects and methods

### *Subjects*

The patients and controls were participating in an ongoing, population-based, case-control interview study. We decided in advance to confine the analysis of hormones and SHBG in the serum to the first 100 cases included and the same number of controls. The study base consisted of all males under the age of 80 years born in Sweden and living in one county (Örebro). The source population comprised around 270,000 individuals in 1988 (The Cancer Registry, 1991). In this population all males suspected of having prostatic cancer are referred to either of the three hospitals in the county for further diagnostic work-up and treatment.

Eligible for the study were all patients with a newly diagnosed, cytologically and/or histologically confirmed prostatic cancer. Of the 100 patients included, 22 had a cancer first detected at histopathologic examination of the surgical specimen removed at TUR ($T_0$); all others had palpable tumours

Correspondence: S.-O. Andersson, Department of Urology, Örebro Medical Center Hospital, S-701 85 Örebro, Sweden.
Received 8 October 1992; and in revised form 21 January 1993.

($T_2$–$T_4$). In all patients the tumour was graded and staged in accordance with the WHO and UICC classifications, respectively (International Histological Classification, 1980; UICC, 1978). The presence of metastases was assessed by skeletal scintigraphy. On the basis of the TNM classification, the patients were classified into one of three mutually exclusive groups, namely a group with localised (T0-2,NX,M0), locally advanced (T3-4,NX,M0) or generalised (T0-4,NX,M1) disease.

Male controls were frequency-matched for age (<50, 50–59, 60–69, 70–79) and randomly selected every 3 months from the county population register. All potential controls underwent clinical examination by one of the investigators (S-O. A.) to rule out the presence of a palpable tumour in the prostate. Persons with a palpable nodule and/or an elevated level (>10 $\mu g\ l^{-1}$) of prostate specific antigen (PSA) in the serum were investigated further by ultrasound-guided biopsies before being definitely accepted as a control.

Blood sample from 100 (86% of all eligible) cases and 100 (76% of all invited) controls were drawn – without fasting – between 08.00–10.00 a.m. and before digital rectal examination of the prostate was performed. The samples were centrifuged and stored at −70°C until assayed. For practical reasons the assays were performed on two occasions (50 cases/50 controls on each occasion). The samples were analysed for luteinising hormone (LH), follicle-stimulating hormone (FSH), estradiol, testosterone (total and free), androstenedione and SHBG. Body mass index (BMI) was calculated as weight (kg)/height (m)$^2$. Seven of the cases had to be excluded from the analysis as they had already received treatment before the blood samples were drawn and two controls proved to have a prostatic cancer and were excluded; hence 93 cases and 98 controls were included in the analysis.

In order to minimise errors due to bias in the analytical method, the patient samples were analysed in random order and every second sample, which was also taken in random order, was a control.

### *Serum hormone assays*

*LH* in 25 μl serum (S-LH) was measured with a time-resolved Sandwich fluoroimmunoassay (Delfia hLH Spec; Pharmacia-Wallac Oy, Turku, Finland). In this assay two different mouse monoclonal antibodies were used, directed against two different sites on the β subunit. One was immobilised onto the walls of microtiter plates and the other was in solution and labelled with europium chelate. The values were expressed in $\mu g\ l^{-1}$ and a highly purified human LH (hLH) preparation was used as a standard. One microgram corresponded to 6.6 IU of the Second International Reference Preparation (2nd IRP) of pituitary FSH/LH (78/594). The reference range for healthy men was 0.2–1.6 $\mu g\ l^{-1}$, and the minimal detection limit was 0.007 $\mu g\ l^{-1}$. The within- and between-assay coefficients of variation were 2.6% and 3.5% respectively.

*FSH* in 25 μl serum (S-FSH) was measured with a time-resolved Sandwich fluoroimmunoassay (Delfia hFSH; Pharmacia-Wallac Oy, Turku, Finland). Two different mouse monoclonal antibodies were used. One was directed against the β subunit of hFSH and immobilised onto the walls of microtiter wells. The other was in solution, labelled with europium chelate, and specific for the α subunit. The values were expressed in $\mu g\ l^{-1}$ and a highly purified hFSH preparation was used as a reference standard. One μg corresponded to 4.7 IU of the 2nd IRP of pituitary FSH/LH (78/594). The reference range for healthy men was 0.25–2.5 $\mu g\ l^{-1}$, and the minimal detection limit was 0.007 $\mu g\ l^{-1}$. The within- and between-assay coefficients of variation were 2.5% and 3.6% respectively.

*Estradiol-17β* in 25 μl serum (S-E2) was measured with a time-resolved solid-phase fluoroimmunoassay (Delfia Estradiol; Pharmacia-Wallac Oy, Turku, Finland), based on competition between europium-labelled estradiol and sample estradiol for polyclonal anti-estradiol antibodies (derived from the rabbit). A second antibody, directed against rabbit IgG, is immobilised onto the walls of microtiter plates, and bind the IgG-estradiol complex. The cross-reactivity for estrone at an inhibition level of 50% was 1.5%. The reference range for healthy men was from non-detectable to 150 pmol $l^{-1}$. The minimal detection limit was about 20 pmol $l^{-1}$. The within-assay coefficient of variation was <10% for values >50 pmol $l^{-1}$ and about 7% at 150 pmol $l^{-1}$.

*Testosterone* (total) in 50 μl serum (S-T) was measured with a solid-phase radioimmunoassay (Coat-A-Count Total Testosterone; Diagnostic Products Corporation, Los Angeles, USA) based on competition between $^{125}$I-labelled testosterone and sample testosterone for polyclonal anti-testosterone antibodies immobilised to the wall of a polypropylene tube. The cross-reactivity with dihydrotestosterone was less than 5%. The reference range for healthy men was 10–45 nmol $l^{-1}$, and the minimal detection limit was 0.07 nmol $l^{-1}$. The within- and between-assay coefficients of variation were 5% and 8% respectively.

*Androstenedione* in 0.25 μl serum (S-ASD) was measured with a radioimmunoassay (Androstenedione Radioimmunoassay Kit, Amersham International, Amersham, UK) based on competition between $^{125}$I-labelled androstenedione and sample androstenedione for polyclonal anti-androstenedione antibodies. The separation of free and bound antigen was achieved by the use of an accelerated double antibody system followed by centrifugation. The reference range for healthy men was 2.0–9.3 nmol $l^{-1}$. The minimal detection limit was 0.15 nmol $l^{-1}$. The within- and between-assay coefficients of variation were 3% and 7% respectively.

*Albumin* in 3 μl serum (S-Albumin) was measured with an automated analysis Hitachi System 717, using bromocresol green as a reagent. The results were expressed in g $l^{-1}$. The reference range was 36–48 g $l^{-1}$, and the between-assay variation was 2%.

*SHBG* in 0.25 μl serum (S-SHGB) was measured with a time-resolved Sandwich fluoroimmunoassay (Delfia SHBG; Pharmacia-Wallac Oy, Turku, Finland). In this assay, the SHBG was bound to polyclonal rabbit anti-SHGB antibodies immobilised onto the walls of microtitration strip wells. Monoclonal mouse anti-SHBG antibodies labelled with europium chelate was then bound to the SHBG. The reference range for healthy men was 1.0–9.3 mg $l^{-1}$. The minimal detection limit was 0.05 mg $l^{-1}$. The within- and between-assay coefficients of variation were 3.5% and 6%, respectively.

*Free testosterone* in serum (S-ft) was calculated by a formula derived from the law of mass action, assuming two binding systems, T-SHBG and T-albumin (Södergård *et al.*, 1982). The fixed values used for the binding affinity constants for T-SHBG and T-albumin were $5.97 \times 10^8\ M^{-1}$ and $4.06 \times 10^4\ M^{-1}$, respectively. The values for S-T (total), S-SHBG and S-albumin were those determined experimentally in every sample. One microgram of SHBG = 10.75 nmol. One gram of albumin = 15,400 nmol.

### *Statistical methods*

The serum hormone and SHBG levels in cases and controls were compared by standard *t* tests. Comparisons after adjustment for age were made by standard multiple regression analyses. In the analyses of the relation between hormone level and the risk of developing cancer, the logistic regression model was used. This model assumes that the logarithm of the odds of developing prostatic cancer is a linear function of the explanatory variables (hormone level and age). By considering the hormone variables in categorised form, nonlinear effects were also allowed. The model was estimated by the maximum likelihood method. From the estimated parameters of the model, the odds ratios associated with different variables and categories were computed.

In the analyses of the influence of tumour stage, tumour grade and age on hormone values, the standard regression model was used. The estimated parameters of this model (Table IV) show the difference between a certain category of an explanatory variable and the reference category of the variable (these were localised disease, high grade and age under 70) after adjustment for the effects of other variables. The same basic model was used in the univariate analyses of the corresponding variables. This yielded the $P$ values shown in Table III.

The results are reported with the hormone variables in logarithmic form. Analyses were also carried out with the variables in untransformed form. The results of these analyses did not differ greatly from those shown.

### Results

The mean age of the 93 prostatic cancer patients (69.7 years) closely agreed with that of the 98 controls (69.6 years). A comparison of the patients (cases) and controls with respect to the serum hormone levels, SHBG and BMI is shown in Table I. In none of these comparisons did the mean values show any statistically significant difference between the groups. The results after age adjustment were almost identical. A remarkably close agreement was found in particular for total and free testosterone, but also for the other hormones and for SHBG. The only marked difference was in the estradiol level, which was about 16% lower in the cases. The interindividual variations within each group were high for almost all these analyses, as indicated by the standard deviations. A logistic regression analysis, with adjustment for age, further confirmed the similarity in mean serum hormone levels between cases and controls.

Table II shows the age-adjusted odds ratio and 95% confidence interval. Each hormone was analysed both as a logarithmic continuous variable and after categorisation into tertiles based on values in the controls, using the lowest tertile as a reference group. No statistically significant difference was found between cases and controls for any of the hormones or for SHBG (Table II). For testosterone, the hormone of primary interest, the odds ratio for the highest compared with the lowest tertile was 0.97, whereas the mean values of these tertiles differed about 2.4-fold. A similar magnitude of variation in the serum concentrations and a similar lack of association with case-control status was noted for free testosterone. There was some evidence of a decreasing risk with increasing serum levels of estradiol and SHBG, but the effect was not statistically significant. Similarly, analyses of each hormone and SHBG in continuous form did not reveal any statistically significant difference between cases and controls; the $P$ values were 0.29 (estradiol) or higher (data not shown).

**Table I** Mean serum concentrations, with standard deviations (SD), of LH, FSH, total and free testosterone, estradiol, androstenedione, SHBG, mean BMI with SD and mean age in 93 patients with prostatic cancer and in 98 population controls

| | *Cases* | | *Controls* | | |
|---|---|---|---|---|---|
| *Factor* | *Mean* | *(SD)* | *Mean* | *(SD)* | *P-value*[a] |
| LH ($\mu$G L$^{-1}$) | 0.917 | (0.665) | 0.968 | (0.832) | 0.70 |
| FSH ($\mu$g l$^{-1}$) | 1.777 | (1.929) | 1.896 | (2.234) | 0.83 |
| Total testosterone (nmol l$^{-1}$) | 15.84 | (6.75) | 15.97 | (6.27) | 0.59 |
| Free testosterone (nmol l$^{-1}$) | 0.295 | (0.111) | 0.293 | (0.103) | 0.86 |
| Estradiol (pmol l$^{-1}$) | 66.2 | (41.4) | 79.0 | (101.4) | 0.30 |
| Androstenedione (nmol l$^{-1}$) | 6.08 | (2.24) | 5.86 | (1.70) | 0.67 |
| SHBG (mg l$^{-1}$) | 4.86 | (2.21) | 5.06 | (2.46) | 0.48 |
| BMI | 26.3 | (3.2) | 25.8 | (2.9) | 0.09 |
| Age | 69.7 | | 69.6 | | |

[a] $t$-test on log values.

**Table II** Odds ratios (OR), adjusted for age with a 95% confidence interval (CI) for each factor analysed as a categorised variable

| | | *Tertiles* | | | |
|---|---|---|---|---|---|
| *Factor* | | *Range* | *Mean* | *OR* | *95% CI* |
| LH | | <0.565 | 0.405 | 1.00 | ref |
| (mg l$^{-1}$) | | 0.565–0.945 | 0.740 | 0.91 | 0.45–1.85 |
| | | >0.945 | 1.683 | 0.88 | 0.43–1.81 |
| FSH | | <0.865 | 0.601 | 1.00 | ref |
| (mg l$^{-1}$) | | 0.865–1.565 | 1.125 | 1.57 | 0.77–3.18 |
| | | >1.565 | 3.720 | 1.19 | 0.57–2.47 |
| Testosterone | | | | | |
| (nmol l$^{-1}$) | total | <12.15 | 9.77 | 1.00 | ref |
| | | 12.15–17.60 | 14.53 | 1.18 | 0.58–2.38 |
| | | >17.60 | 23.05 | 0.97 | 0.49–1.94 |
| | free | <0.243 | 0.195 | 1.00 | ref |
| | | 0.243–0.327 | 0.281 | 1.51 | 0.75–1.11 |
| | | >0.327 | 0.407 | 1.21 | 0.60–2.42 |
| Estradiol | | <42.5 | 25.9 | 1.00 | ref |
| (pmol l$^{-1}$) | | 42.5–84.5 | 64.2 | 0.85 | 0.42–1.71 |
| | | >84.5 | 127.5 | 0.61 | 0.30–1.25 |
| Androstenedione | | <5.10 | 4.08 | 1.00 | ref |
| (nmol l$^{-1}$) | | 5.10–6.45 | 5.78 | 1.06 | 0.53–2.13 |
| | | >6.45 | 8.05 | 0.88 | 0.44–1.77 |
| SHBG | | <3.75 | 2.97 | 1.00 | ref |
| (mg l$^{-1}$) | | 3.75–5.55 | 4.57 | 0.90 | 0.45–1.82 |
| | | >5.55 | 7.33 | 0.76 | 0.37–1.56 |

The possibility that serum concentrations of hormones or SHBG might be associated with the tumour stage, tumour grade or age was also analysed (Table III). Increasing values of LH ($P = 0.005$) and FSH ($P = 0.007$) with advancing tumour stage were found. A similar, but nonsignificant, tendency toward increasing values of LH and FSH with a decreasing grade of differentiation was also evident. Patients over the age of 70 years tended to have higher values of LH, FSH and SHBG than younger patients, and this difference was statistically significant for FSH ($P = 0.02$) and SHBG ($P = 0.02$). Among the controls also, the LH, FSH and SHBG values tended to be higher in the over-70 age group (data not shown). In contrast, the steroid hormone levels were seemingly unrelated to stage, grade or age of the patient (Table III).

Thus, only LH, FSH and SHBG showed a statistically significant relation to tumour stage, tumour grade or age. These factors were studied simultaneously in multivariate analyses with each hormone as the dependent variable with the aim of finding independent determinants of serum concentrations and to quantify the strength of the association (Table IV). Increasing values for LH and FSH with increasing tumour burden were found ($P = 0.05$ and $P < 0.05$ respectively). The mean logarithmic values for LH and FSH were 0.50 and 0.69 units respectively, and were higher in generalised than in localised disease. After adjustment for tumour stage (and age), no significant association was seen between tumour grade and LH or FSH. Patients above 70 years of age had slightly higher values of LH, FSH and SHBG than younger patients; the logarithmic value for SHBG was 0.21 units higher than in younger patients ($P < 0.05$).

### Discussion

This study was carried out on a consecutive sample of patients with newly diagnosed prostatic cancer in a strictly defined population. In contrast to several previous studies (Harper *et al.*, 1976; Bartsch *et al.*, 1977*b*; Hammond *et al.*, 1978; Habib, 1980; Saroff *et al.*, 1980; Høisaeter *et al.*, 1982; Zumoff *et al.*, 1982; Ranikko & Adlercreutz, 1983; Hulka *et al.*, 1987), the control group was population-based and prostate cancer was ruled out by digital rectal examination and serum analysis for prostate-specific antigen. Serum samples were drawn, stored and analysed according to highly standar-

**Table III** Mean serum concentrations, with standard deviations (SD), of LH, FSH, total and free testosterone, estradiol, androstenedione and SHBG by tumour stage, tumour grade and age in 93 patients with prostatic cancer

| *Factor* | | *Stage* *T0-2NXM0* | *T3-4NXM0* | *T0-4NXM1* | *Grade* *High* | *Medium* | *Low* | *Age* $<70$ | $>70$ |
|---|---|---|---|---|---|---|---|---|---|
| LH | Mean | 0.77 | 1.02 | 1.26[a] | 0.87 | 0.93 | 1.22 | 0.79 | 1.03 |
| ($\mu g\ l^{-1}$) | SD | 0.51 | 0.80 | 0.71 | 0.63 | 0.69 | 0.81 | 0.58 | 0.72 |
| FSH | Mean | 1.42 | 2.06 | 2.53[b] | 1.69 | 1.77 | 2.37 | 1.30 | 2.20[c] |
| ($\mu g\ l^{-1}$) | SD | 1.21 | 2.71 | 1.76 | 1.38 | 2.72 | 1.96 | 1.04 | 2.40 |
| Testosterone | Mean | 15.4 | 17.4 | 13.9 | 15.3 | 17.8 | 12.2 | 15.1 | 16.5 |
| ($nmol\ l^{-1}$) | SD | 5.5 | 8.1 | 7.3 | 5.0 | 9.0 | 6.5 | 5.5 | 7.7 |
| Testosterone, free | Mean | 0.29 | 0.32 | 0.26 | 0.29 | 0.33 | 0.24 | 0.29 | 0.30 |
| ($nmol\ l^{-1}$) | SD | 0.088 | 0.141 | 0.108 | 0.077 | 0.151 | 0.123 | 0.081 | 0.133 |
| Estradiol | Mean | 63.6 | 73.2 | 58.8 | 65.8 | 65.4 | 72.3 | 60.7 | 71.1 |
| ($pmol\ l^{-1}$) | SD | 39.8 | 42.1 | 46.9 | 41.8 | 41.1 | 44.6 | 35.8 | 45.6 |
| Androstenedione | Mean | 6.03 | 6.25 | 5.88 | 6.08 | 6.30 | 5.28 | 6.33 | 5.86 |
| ($nmol\ l^{-1}$) | SD | 2.21 | 2.38 | 2.20 | 1.97 | 2.85 | 1.47 | 2.42 | 2.07 |
| SHBG | Mean | 4.70 | 5.11 | 4.88 | 4.75 | 5.16 | 4.54 | 4.44 | 5.23[d] |
| ($mg\ l^{-1}$) | SD | 2.36 | 1.96 | 2.29 | 1.77 | 3.02 | 1.49 | 2.45 | 1.91 |

[a]$P = 0.005$ compared with stage T0-2NXM0. [b]$P = 0.007$ compared with stage T0-2NXM0. [c]$P = 0.02$ compared with age $<70$. [s]$P = 0.02$ compared with age $<70$.

dised routines. Differential misclassification between cases and controls is therefore unlikely, and we consider the internal validity of the study as high. Moreover, the numbers of cases and controls should imply a statistical power that is higher than in most published investigations (Table V). In addition, when analyses are based on only one sample, short-term, intraindividual variations in hormone concentrations are not large enough to conceal, to any appreciable extent, any differences that may be present between groups (Dai *et al.*, 1981).

Our main finding was the absence of an association between prostatic cancer and pituitary hormones, sex steroid hormones, or SHBG. Indeed, for most variables the similarity in mean values (Table I) and the lack of a trend after categorisation into tertiles (Table II) was remarkable. We noted with special interest that the mean serum concentrations of total and free testosterone – the latter the biologically active form – were virtually identical in cases and controls; and further that a recently reported association between androstenedione and prostate cancer (Barrett-Connor *et al.*, 1990) was not supported by our data.

The main results in the literature of comparisons regarding pituitary and sex steroid hormone levels between patients with prostatic cancer and controls are presented in Table V. The great disparity in the criteria for selection of controls, the small numbers of subjects, and the differences in study design should be noted. These inconsistencies may partly explain the varying results.

**Table IV** The influence of stage, grade and age on selected hormone variables in 93 patients with prostatic cancer. Multivariate analyses. Parameter estimates with standard errors in parentheses. All hormone variables are given in logarithmic form

| *Factor* | *LH* | *FSH* | *SHBG* |
|---|---|---|---|
| Const | − 0.50 | 0.05 | 1.38 |
| | (0.11) | (0.12) | (0.09) |
| $T_{3-4}N_xM_0$ | 0.26 | 0.33 | 0.05 |
| | (0.16) | (0.17) | (0.10) |
| $T_{0-4}N_xM_1$ | 0.50[a] | 0.69[B] | − 0.04 |
| | (0.25) | (0.28) | (0.17) |
| Diff grade Medium | − 0.09 | − 0.42[b] | − 0.03 |
| | (0.16) | (0.18) | (0.11) |
| Diff grade Low | 0.13 | − 0.06 | − 0.03 |
| | (0.28) | (0.31) | (0.18) |
| Age $\geqslant 70$ | 0.13 | 0.27 | 0.21[b] |
| | (0.14) | (0.16) | (0.09) |
| $R^2$ | 0.1103 | 0.1666 | 0.0668 |

[a]$P = 0.051$. [b]$P < 0.05$.

What conclusions can be drawn from the results of this study regarding the causal role of hormones in the etiology of prostatic cancer? Firstly, serum hormone levels as determined at the time of diagnosis are seemingly not related to the occurrence of prostatic cancer. However, this does not preclude the possibility that men who later develop prostatic cancer may differ in their serum hormone level at younger ages, when the malignant transformation is initiated, and that these differences diminish with age. In the United States, the incidence of and mortality from cancer of the prostate is substantially higher in blacks than in whites (Greenwald, 1982). Black college students have been reported to have higher total and free testosterone levels than whites (Ross *et al.*, 1986).

According to a rival theory, hormones may be important in the etiology in terms of individual susceptibility at the cellular level, for example the receptor function or hormone metabolism may be altered in those who develop prostatic cancer. In one study an increased production and increased metabolic clearance rate of testosterone was found even in patients whose serum testosterone levels did not differ from those in controls (Meikle *et al.*, 1989). In a recent study the serum testosterone concentrations in young adult Japanese men were compared with those in young adult whites and blacks from the US, but no significant differences were found. However, the activity of 5-α-reductase was found to be significantly lower in the Japanese than in the US men (Ross *et al.*, 1992). These findings suggest that hormone metabolism may be of importance in the etiology of prostate cancer. A great disparity between the concentrations of androgens in prostate tissue and serum has also been reported (Habib, 1980). A third theory is that hormones play a permissive role in the etiology of prostatic cancer, but that variations within the normal range are not associated with the risk of developing clinical disease. This need not rule out their importance, however, in the progression, growth and clinical course of the disease (Ishikawa *et al.*, 1989).

The reason for the higher serum levels of LH and FSH in patients with more advanced tumours is not clearly understood. These changes may conceivably be secondary to the (non-significant) decrease in the serum level of testosterone in patients with more advanced cancer. It is not known, however, whether a low serum testosterone level is a consequence or a cause of advanced disease.

In conclusion, this study has shown that at the time of diagnosis there is no difference in the serum levels of sex steroid or pituitary hormones or of SHBG between patients with prostatic cancer and healthy age-matched controls.

**Table V** Main results of case-control and cohort studies with comparisons of serum hormone levels between patients with prostatic cancer and controls

| *Author* | *Number* *Cases* | *Controls* | *Type[a] of controls* | *Results[b]* *Higher in cases* | *No difference* | *Lower in cases* |
|---|---|---|---|---|---|---|
| Young & Kent, 1968 | 28 | 55 | C + E | | T | |
| Habib *et al*., 1976 | 10 | 10 | D | | T,DGT,A | |
| Harper *et al*., 1976 | 33 | 76 | B + D | | T,A,$E_2$,FSH,LH | |
| Bartsch *et al*., 1977*a* | 29 | 38 | B + E | | $E_1$,$E_2$,FSH,T | DHT,LH |
| Bartsch *et al*., 1977*b* | 33 | 38 | E | | T,DHT,$E_1$,$E_2$,SHBG | |
| Hammond *et al*., 1978 | 11 | 97 | D + E | | T,$E_2$,A | |
| Ghanadian *et al*., 1979 | 33 | 42 | E | T | DHT | |
| Jackson *et al*., 1980 | US black 108 | 183 | B | T,$E_1$ | | |
| | Nigerians 65 | 70 | | | $E_1$ | T |
| Jacobi *et al*., 1980 | 73 | 72 | D + E | | T,P | |
| Saroff-Kirdani *et al*., 1980 | 97 | 26 | D + E | | | T,DHT |
| Ahluwalia *et al*., 1981 | US black 170 | unknown | E | T,$E_1$ | DHT,$E_2$ | |
| | Nigerians 55 | | | | DHT,$E_2$ | T |
| Drafta *et al*., 1982 | 32 | 108 | D + E | T | | $E_1$,$E_2$ |
| Hill *et al*., 1982 | 21 | 19 | D + E | $E_1$ | $E_2$ | T,A |
| Høisaeter *et al*., 1982 | 100 | | E | SHBG | T,$E_2$,FSH,LH | |
| Meikle *et al*., 1982 | 126 | 54 | E | | $E_2$,SHBG | T |
| Zumoff *et al*., 1982 | 16 | 36 | E | $E_1$ | LH,FSH,P | T |
| Rannikko *et al*., 1983 | 30 | 50 | D + E | SHBG | T,$E_2$,P | TF |
| Levell *et al*., 1985 | 80 | 71 | D | | TF,T (>75 yrs) | TF,T (<65 yrs) |
| Hulka *et al*., 1987 | 35 | 203 | D | | T,$E_2$ | |
| Nomura *et al*., 1988 | 98 | 98 | Cohort 6860 males | | T,DHT,$E_1$,$E_2$,SHBG | |
| Hsing *et al*., 1989 | 103 | 103 | A | | T,DHT,$E_1$,$E_2$,P,FSH,LH | |
| Meikle *et al*., 1989 | 38 | 22 | E | | $E_2$,T,LH,FSH | |
| Barrett-Connor, 1990 | 57 | | Cohort 1008 males | A | T,$E_1$,$E_2$,SHBG | |

[a]A = population controls, B = hospital controls, C = patients with other cancers, D = patients with benign prostatic hyperplasia, E = healthy but not population-based controls. [b]A = Androstenedione, D = Dihydrotestosterone, $E_1$ = Estrone, $E_2$ = Estradiol, SHBG = Sex-hormone-binding globulin, FSH = Follicle-stimulating hormone, LH = Luteinizing hormone, P = Prolactin, T = Testosterone, TF = Free testosterone.

Future investigations on the possible role of hormones in the etiology of prostatic cancer should therefore probably be focused on earlier ages, or on intracellular metabolic changes.

This work was supported by grants from Örebro County Council Research Committee and The Swedish Cancer Society.

### References

AHLUWALIA, B., JACKSON, M.A., JONES, G.W., WILLIAMS, A.O., RAO, M.S. & RAJGURU, S. (1981). Blood hormone profiles in prostate cancer patients in high-risk and low-risk populations. *Cancer*, **48**, 2267–2273.

BARRACK, E.R., BUJNOVSKY, P. & WALSH, P.C. (1983). Subcellular distribution of androgen receptors in human normal, benign hyperplasia and malignant prostatic tissues: characterization of nuclear salt-resistant receptors. *Cancer Res*,. **43**, 1107–1116.

BARRETT-CONNOR, E., GARLAND, C., MCPHILLIPS, J.B., KHAW, K.T. & WINGARD, D.L. (1990). A prospective, population-based study of androstenedione, estrogens and prostatic cancer. *Cancer Res*., **50**, 169–173.

BARTSCH, W., STEINS, P. & BECKER, H. (1977*a*). Hormone blood levels in patients with prostatic carcinoma and their relation to the type of carcinoma growth differentiation. *Eur. Urol*., **3**, 47–52

BARTSCH, W., HORST, H.-J., BECKER, H. & NEHSE, G. (1977*b*). Sex hormone binding globulin capacity, testosterone, 5-alfa-dihydrotestosterone, oestradiol and prolactin in plasma of patients with prostatic carcinoma under various types of hormonal treatment. *Acta Endocrinol*,. **85**, 650–664.

COFFEY, D.S. & ISAACS, J.T. (1981). Control of prostate growth. *Urology*, Suppl. **3**, 17–24.

DAI, W.S., KULLER, L.H., LAPORTE, R.E., GUTAI, P,. FALVO-GERARD, L. & GAGGIULA, A. (1981). The epidemiology of plasma testosterone levels in middle-aged men. *Am. J. Epidemiol*., **114**, 804–816.

DE VITA, V.T., HELLMAN, S. & ROSENBERG, S.A. (1982). *Cancer: Principles and practise of Oncology*. Lippincott: Philadelphia.

DRAFTA, D., PROCA, E., ZAMFIR, V., SCHINDLER, A.E., NEACSU, E. & STROE, E. (1982). Plasma steroids in benign prostatic hypertrophy and carcinoma of the prostate. *J. Steroid Biochem*., **17**, 689–693.

GHANADIAN, R., PUAH, C.M. & O'DONOGHUE, E.P.N. (1979). Serum testosterone and dihydrotestosterone in carcinoma of the prostate. *Br. J. Cancer*, **39**, 696–699.

GREENWALD, P. Prostate. In: Schottenfeld, D. & Fraumeni, J.F., Jr. (eds) (1982). *Cancer Epidemiology and Prevention*, pp. 9138–9146. Saunders: Philadelphia.

HABIB, F.K. (1980). Evaluation of androgen metabolism studies in human prostate cancer: correlation with zinc level. *Prevent. Med*., **9**, 650–656.

HABIB, F.K., ODOMA, S., BUSUTTIL, A. & CHISHOLM, G.D. (1986). Androgen receptors in cancer of the prostate – correlation with the stage and grade of the tumor. *Cancer*, **57**, 2351–2356.

HAMMOND, G., KONTTURI, M., VIHKO, P. & VIHKO, R. (1978). Serum steroids in normal males and patients with prostatic diseases. *Clin. Endocrinol*., **9**, 113–121.

HARPER, M.E., PEELING, W.B., COWLEY, T., BROWNSEY, B.G., PHILLIPS, M.E.A., GROOM, G., FAHMY, D.R. & GRIFFITHS, K. (1976). Plasma steroid and protein hormone concentrations in patients with prostate cancer before and during oestrogen therapy. *Acta Endocrinol. (Copenh)*., **81**, 409–426.

HILL, P., WYNDER, E.L., GARBACZEWSKI, L. & WALKER, A.R.P. (1982). Effect of diet on plasma and urinary hormones in South African black men with prostatic cancer. *Cancer Res*., **42**, 3864–3869.

HØISAETER, P.A., HAUKAAS, S., BAKKE, A., HOIEM, L., SEGADAL, E. & THORSEN, T. (1982). Blood hormone levels related to stages and grades of prostatic cancer. *Prostate*, **3**, 375–381.

HSING, A. & COMSTOCK, G. (1989). Serum hormone and risk of subsequent prostate cancer. *Am. J. Epidemiol*., **130**, 829.

HUGGINS, C. & HODGES, C.V. (1941). Studies on prostatic cancer. II. The effects of castration on advanced carcinoma of the prostate gland. *Arch. Surg*., **43**, 209–223.

HULKA, B.S., HAMMOND, J.E., DIFERNANDO, G., MICKEY, D.D., FRIED, F.A., CHECKOWAY, H., STUMPF, W.E., BECKMAN, W.C. & CLARK, T.D. (1987). Serum hormone levels among patients with prostatic carcinoma or benign prostatic hypertrophy and clinic controls. *Prostate*, **11**, 171–182.

INTERNATIONAL HISTOLOGICAL CLASSIFICATION OF TUMORS. No 22 (1980). *Histological Typing of Prostate Tumors*. WHO: Geneva.

ISHIKAWA, S., SOLOWAY, M.S., VAN DER ZWAAG, R. & TODD, B. (1989). Prognostic factors in survival free of progression after androgen deprivation therapy for treatment of prostate cancer. *J. Urol.*, **14**, 1139.

JACKSON, M.A., KOVI, J., HESHMAT, M.Y., OGUNMUYIWA, T.A., JONES, G.W., WILLIAMS, A.O., CHRISTIAN, E.C., NKPOSONG, E.O., RAO, M.S., JACKSON, A.G. & AHLUWALIA, B. (1980). Characterization of prostatic carcinoma among blacks: a comparison between a low-incidence area, Ibadan, Nigeria, and a high-incidence area, Washington DC. *Prostate*, **1**, 185–205.

JACOBI, G.H., RATHGEN, G.H. & ALTWEIN, J.E. (1980). Serum prolactin and tumors of the prostate: unchanged, basal levels and lack of correlation to serum testosterone. *J. Endocrinol.*, **3**, 15–18.

LEVELL, M.J., ROWE, E., GLASHAN, R.W., PIDCOCK, N.B. & SIDDALL, J.K. (1985). Free testosterone in carcinoma of the prostate. *Prostate*, **7**, 363–367.

MEIKLE, A.W. & STANISH, W. (1982). Familial prostatic cancer risk and low testosterone. *J. Clin. Endocrinol. Metabol.*, **54**, 1104–1108.

MEIKLE, A.W., SMITH, J.A. & STRINGHAM, J.D. (1989). Estradiol and testosterone metabolism and production in men with prostatic cancer. *J. Steroid Biochem.*, **33**, 19–24.

NOBLE, R.L. (1977). The development of prostatic adenocarcinoma in Nb rats following prolonged sex hormone administration. *Cancer Res.*, **37**, 1929–1933.

NOMURA, A., HEILBRUN, L.K., STEMMERMANN, G.N. & JUDD, H.J. (1988). Prediagnostic serum hormones and the risk of prostate cancer. *Cancer Res.*, **48**, 3515–3517.

RANIKKO, F.S. & ADLERCREUTZ, H. (1983). Plasma estradiol, free testosterone, sex hormone binding globulin capacity and prolactin in benign prostatic hyperplasia and prostatic cancer. *Prostate*, **4**, 223–229.

ROSS, R., BERNSTEIN, L., JUDD, H., HANISCH, R., PIKE, M. & HENDERSON, B. (1986). Serum testosterone levels in healthy young black and white men. *J. Natl Cancer Inst.*, **76**, 45–48.

ROSS, R., BERNSTEIN, L., LOBO, R., SHIMIZU, H., STANCZYK, F., PIKE, M. & HENDERSON, B. (1992). 5-Alpha-reductase activity and risk of prostate cancer among Japanese and US white and black males. *Lancet*, **339**, 887–889.

SAROFF, J., KIRDANI, R.Y., MING CHU, T., WAJSMAN, Z. & MURPHY, G. (1980). Measurements of prolactin and androgens in patients with prostatic diseases. *Oncology*, **37**, 46–52.

SÖDERGÅRD, R., BÄCKSTRÖM, T., SHAN BHAG, V. & CARSTENSEN, H. (1982). Calculation of free and bound fractions of testosterone and estradiol-17β to human plasma proteins of body temperature. *J. Steroid Biochem.*, **16**, 801–810.

THE CANCER REGISTRY (1991). *Cancer Incidence in Sweden 1988*. National Board of Health and Welfare: Stockholm.

UICC. UNION INTERNATIONALE CONTRE LE CANCER (1978). *TNM Classification of Malignant Tumours*. Third edition. International Union against Cancer: Geneva.

YOUNG, H. & KENT, J. (1968). Plasma testosterone levels in patients with prostatic carcinoma before and after treatment. *J. Urol.*, **99**, 788–792.

ZUMOFF, B., LEVIN, J., STRAIN, G.W., ROSENFELD, R.S., O'CONNOR, J., FREED, S.Z., KREAM, J., WHITMORE, W., FUKUSHIMA, D. & HELLMAN, L. (1982). Abnormal levels of plasma hormones in men with prostate cancer: evidence toward a 'two-disease' theory. *Prostate*, **3**, 579–588.